# Supplementary material for: Polyketide synthase gene clusters in the ericoid mycorrhizal fungus Oidiodendron maius: genome-wide prediction, expression profiling, and functional characterization of knockout mutants
Source: Front Plant Sci. 2026 Sep 16;17:1924430. doi: 10.3389/fpls.2026.1924430 (PMC13623903; doi:10.3389/fpls.2026.1924430)
Supplement: Supplementary file 1 [file SupplementaryFile1.zip › Supplementary Materials/Supplementary Folder 1.zip!/Oidma1_AssemblyScaffolds_Repeatmasked/knownclusterblast/cluster7/jgi.p_Oidma1_175489_mibig_hits.html]

| MIBiG Protein | Description | MIBiG Cluster | MiBiG Product | % ID | % Coverage | BLAST Score | E-value |
| --- | --- | --- | --- | --- | --- | --- | --- |
| PSCXE2 |  | BGC0001325 | Noscapine\_biosynthetic\_gene\_cluster. | 32 | 43.3333333333 | 61 | 3e-09 |
| PSCXE1 |  | BGC0001325 | Noscapine\_biosynthetic\_gene\_cluster. | 34 | 32.4242424242 | 60 | 5.1e-09 |
| AFR69339.1 | esterase/lipase\_SpiI | BGC0001045 | Spiruchostatin\_biosynthetic\_gene\_cluster | 27 | 56.9696969697 | 53 | 6.3e-07 |
| ABC38006.1 | lipase/esterase | BGC0000964 | Burkholdac\_biosynthetic\_gene\_cluster | 26 | 58.7878787879 | 50 | 4.1e-06 |
| ABP57753.1 | DepI | BGC0000993 | FK228\_biosynthetic\_gene\_cluster | 26 | 60.0 | 50 | 5.3e-06 |
